# Functional annotation of the human retinal pigment epithelium transcriptome

**DOI:** 10.1186/1471-2164-10-164

**Published:** 2009-04-20

**Authors:** Judith C Booij, Simone van Soest, Sigrid MA Swagemakers, Anke HW Essing, Annemieke JMH Verkerk, Peter J van der Spek, Theo GMF Gorgels, Arthur AB Bergen

**Affiliations:** 1 Department of Molecular Ophthalmogenetics, Netherlands Institute for Neuroscience (NIN), an institute of the Royal Netherlands Academy of Arts and Sciences (KNAW), Meibergdreef 47, 1105 BA Amsterdam, the Netherlands (NL); 2 Department of Bioinformatics, Erasmus Medical Center, 3015 GE Rotterdam, the Netherlands; 3 Department of Genetics, Erasmus Medical Center, 3015 GE Rotterdam, the Netherlands; 4 Department of Clinical Genetics, Academic Medical Centre Amsterdam, the Netherlands

## Abstract

**Background:**

To determine level, variability and functional annotation of gene expression of the human retinal pigment epithelium (RPE), the key tissue involved in retinal diseases like age-related macular degeneration and retinitis pigmentosa. Macular RPE cells from six selected healthy human donor eyes (aged 63–78 years) were laser dissected and used for 22k microarray studies (Agilent technologies). Data were analyzed with Rosetta Resolver, the web tool DAVID and Ingenuity software.

**Results:**

In total, we identified 19,746 array entries with significant expression in the RPE. Gene expression was analyzed according to expression levels, interindividual variability and functionality. A group of highly (n = 2,194) expressed RPE genes showed an overrepresentation of genes of the oxidative phosphorylation, ATP synthesis and ribosome pathways. In the group of moderately expressed genes (n = 8,776) genes of the phosphatidylinositol signaling system and aminosugars metabolism were overrepresented. As expected, the top 10 percent (n = 2,194) of genes with the highest interindividual differences in expression showed functional overrepresentation of the complement cascade, essential in inflammation in age-related macular degeneration, and other signaling pathways. Surprisingly, this same category also includes the genes involved in Bruch's membrane (BM) composition. Among the top 10 percent of genes with low interindividual differences, there was an overrepresentation of genes involved in local glycosaminoglycan turnover.

**Conclusion:**

Our study expands current knowledge of the RPE transcriptome by assigning new genes, and adding data about expression level and interindividual variation. Functional annotation suggests that the RPE has high levels of protein synthesis, strong energy demands, and is exposed to high levels of oxidative stress and a variable degree of inflammation. Our data sheds new light on the molecular composition of BM, adjacent to the RPE, and is useful for candidate retinal disease gene identification or gene dose-dependent therapeutic studies.

## Background

The retinal pigment epithelium (RPE) is a multifunctional neural-crest derived cell layer, flanked by the photoreceptor cells on the apical side and the Bruch's membrane (BM)/choroid complex on the basolateral side. Among others, the RPE supplies the photoreceptors with nutrients, regulates the ion balance in the subretinal space and recycles retinal from the photoreceptor cells, which is necessary for the continuation of the visual cycle.[1] It also phagocytoses and degrades photoreceptor outer segments and absorbs light that is projected onto the retina.[1] Finally, the RPE secretes a number of growth factors that maintain the structure and cellular differentiation of the adjacent tissues.[1]

The importance of the RPE in vision is illustrated by the major involvement of this monolayer of cells in genetically determined retinal diseases like age related macular degeneration (AMD) and retinitis pigmentosa (RP).[2] Since the great majority of genes implicated in AMD or RP are expressed in either the RPE or the photoreceptors, the identification of additional genes highly expressed in the RPE may provide valuable clues in the search for new genes involved in retinal disease. [2-6]

Obviously, the functional properties of RPE cells are determined by the genes they express and the proteins they encode. Although the RPE cell is one of the best studied neural cell types, [3-12] large scale assignment of expressed genes to the RPE has been largely dependent on RNA based studies. Assignment of proteins to the RPE has been hampered by its autofluorescence and melanin content. Large-scale RPE related expression studies were performed using cDNA arrays, serial analysis of gene expression (SAGE), expressed sequence tag (EST) analysis, and multiple RT-PCRs. The number of eyes used in these studies ranged from one to fifteen, and the number of genes under investigation from 29 to 30,000. [8-12] While these studies provided valuable information, they were limited in either the number of genes or the number of eyes under investigation, or they lacked specificity due to the tissue sampling method used. Moreover, most or all of these studies focused on the mean gene expression profile of all samples together, rather than documenting potential interindividual differences. [8-12] A robust and specific dataset on RPE expression levels from a substantial number of individuals is lacking and a great deal remains unknown with regard to the interindividual expression differences.

A number of biological processes and cellular functions of genes expressed in the RPE were described in three of the above mentioned studies.[8,10,12] All three identified protein metabolism and signal transduction as an important functional class of genes expressed by the RPE.[8,10,12] Similarly, cell structure,[8,10] cell proliferation,[8,10] gene transcription[10,11] and energy metabolism were described in two out of three studies. Finally, individual studies also identified overrepresentation of membrane proteins,[10] transport or channel proteins,[10] heat shock proteins[10] and vitamin A metabolism.[11] In a recent microarray study we compared RPE gene expression in the macula with the retinal periphery and demonstrated, among other things, consistent differential expression of extracellular matrix genes corresponding with proteins in BM.[13]

The aim of the current study is to describe the gene expression levels and the interindividual variation in gene expression of native human macular RPE cells in a systematic fashion. In addition, we annotate the functions and biological pathways associated with RPE expressed (disease) genes.

To our knowledge this is the first study to present data on (interindividual differences in) human macular RPE gene expression and interindividual differences on a large scale of 22,000 genes, resulting in a further detailed description of the RPE transcriptome.

## Results

RNA from six selected human macular RPE samples was hybridized to six custom made 22 k microarrays enriched for neural transcripts. We functionally annotated and analyzed the data using Rosetta Resolver, the web tool DAVID and Ingenuity software, with regard to gene expression level and variability as well as functional annotation. Furthermore, we specifically looked at the expression levels and variability of retinal disease genes.

### Analysis of gene expression levels (μ_int_)

**Figure 1:**
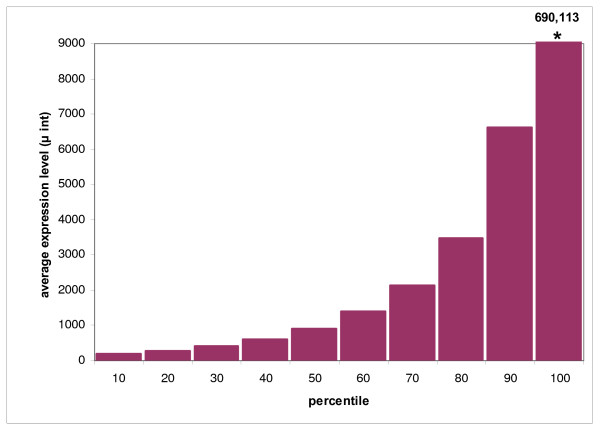
**Distribution of the mean intensity (μ_int_) of all genes across percentile bins of 10 percent**. Each bin contains 2,194 genes. Note that the mean expression level associated with the 100^th ^percentile bin is not displayed fully in this graph due to the height of the expression exceeding the scope of the graph.

The mean expression intensities (μ_int_) ranged from 73 to 690,113 (arbitrary units), (see Additional file 1: Expression level and interindividual variation in all genes on the custom microarray). The distribution of μ_int _across percentile bins of 10 percent of all genes is shown in Figure 1. We used the 90^th^, 50^th ^and 10^th ^percentile of the μ_int _to categorize our data into groups with high (> 90^th^), moderate (50^th^–90^th^), low (10^th^–50^th^) and very low (< 10^th^) expression. We focused our analysis on the biologically most relevant gene groups with high, moderate and low gene expression levels. These categories yielded 2,194 genes with high RPE expression, 8,776 genes with moderate expression and 8,776 genes with low expression. The results of the overrepresentation analysis are presented below, and in Table 1. The overrepresentation analysis of all expressed genes,*irrespective *of their gene expression level (Table 1), did not yield additional functional categories apart from ECM-receptor interaction, and is not presented separately.

**Table 1:** Overrepresented Kegg pathways in macular RPE expressed genes with high, moderate and low expression levels and high or low levels of interindividual variability (coefficient of variation, CV).

		expression level			
		
		all expression levels	high (> 90th perc)	moderate (50th–90th perc)	low (10th–50th perc)
	all CV	ecm-receptor interaction (E)	oxidative phosphorylation (B,E) ribosome (B,E) ATP synthesis (B,E)	phosphatidylinositol signaling system (B,E) aminosugars metabolism (E)	neuroactive ligand receptor interaction (B,E) long-term depression (E) o-glycan biosynthesis (E) calcium signaling pathway (E)
	
CV	high CV	type I diabetes mellitus (B,E) focal adhesion (B,E) cytokine-cytokine receptor interaction (E) complement and coagulation cascades (E) antigen processing and presentation (E) ecm-receptor interaction (E)	antigen processing and presentation (B,E) complement and coagulation cascades (E)	focal adhesion (E) cytokine-cytokine receptor interaction (E)	type I diabetes mellitus (E)
	
	low CV	-	glycosaminoglycan degradation (E)	-	-

Overrepresented pathways were identified with B: a Benjamini-Hochberg corrected p value < 0.001, or E: an Ease score p value < 0.001. Perc: percentile, high CV: > 90^th ^percentile, low CV: < 10^th ^percentile.

#### Genes with high expression levels (μ_int _> 90^th ^percentile, n = 2,194)

**Figure 2:**
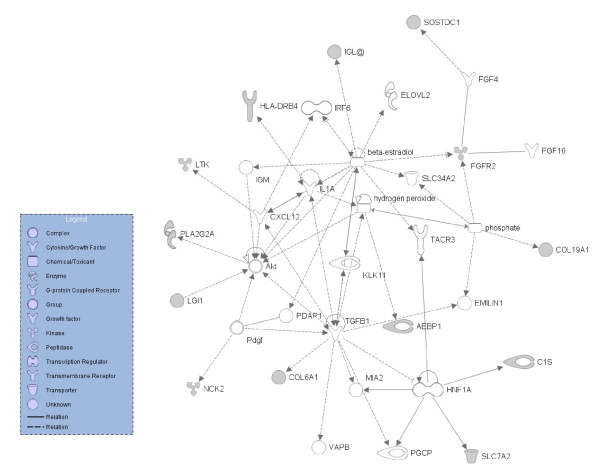
**Ingenuity analysis of the cross section of genes previously identified in RPE studies**[14]**with genes highly expressed in the RPE transcriptome**. The resulting network shows a connectivity chart illustrating biological functions comprising genes, proteins and ligands related to cell-cell signaling and cell-cell interaction. This network contains 13 of the 16 genes entered into the analysis. Filled objects represent the genes entered, empty objects are genes introduced by the ingenuity software creating a connection between the entered genes.

We considered the group of highly expressed genes the most biologically relevant, and, consequently, for this group bioinformatic analysis was more extensive than for other categories. In addition to a Kegg pathway analysis, we also performed an Ingenuity analysis of the overlap between our highly expressed genes and those identified in the literature. Kegg pathway analysis revealed oxidative phosphorylation, ribosome and ATP synthesis as significantly overrepresented pathways (Benjamini-Hochberg p value < 0.001) (Table 1). There was an overlap of 1,407 genes between the highly expressed genes of the RPE transcriptome and the genes identified in retina/RPE genes identified in at least two studies in the literature.[14] Ingenuity analysis of the overlapping genes revealed oxidative phosphorylation as the most significant pathway involved. Comparison of our highly expressed genes to those expressed only in RPE studies (n = 17),[14] showed a clustering of genes in the cell-cell signaling and interaction network (Figure 2).

The thirty most highly expressed RPE genes from our data set are presented in Table 2. Most notably, this list contains two glutamate transporters (*SLC1A2 *[genbank:AF131756] and *SLC17A7 *[genbank: NM_020309])[15], one of which is known to be expressed in the RPE (*SLC17A7 *[genbank: NM_020309])[14] and a gene (*CST3 *[genbank: NM_000099]), that was previously suggested to have an association with AMD,[16,17] with known expression in the RPE.[18] The top thirty list contained three additional genes with known expression in the RPE (*PTGDS *[genbank: NM_000954],[17,19]*TTR *[genbank: NM_000371][14,20] and *HSP90B1 *[genbank: NM_003299])[21] and two genes that play a role in the protection against oxidative stress (*MT1A *[genbank: K01383][22], and *TP53 *[genbank: NM_000546][23]). Finally, we identified a number of genes with a relevant cellular function described in other tissues than the retina, like CLU [NM_001831] (complement system) and ACN9 [NM_020186] (gluconeogenesis).[24,25]

**Table 2:** The thirty most highly expressed genes in macular RPE identified in six different human donors, sorted by intensity in descending order.

gene symbol	Genbank ID	mean intensity μ_int _perc	CV perc	gene name	relevant function
*KIAA0241*	AA205569	99	98	KIAA0241	

*AI003379*	AI003379	99	98	Transcribed locus	

*ACN9*	NM_020186	99	98	ACN9 homolog	gluconeogenesis [25]

*MT1A*	BG191659	99	98	Metallothionein 1A	protection against reactive oxygen species[22]

*SLC17A7*	NM_020309	99	98	Solute carrier family 17 (sodium-dependent inorganic phosphate cotransporter), member 7	glutamate transporter, expressed in RPE[14,15]

*TP53*	NM_000546	99	98	Tumor protein p53	protection against reactive oxygen species[23]

*ELF2*	NM_006874	99	98	E74-like factor 2 (ets domain transcription factor)	

*AI272368*	AI272368	99	95	cDNA clone	

*AA807363*	AA807363	99	79	cDNA clone	

*SERPINB5*	NM_002639	99	98	Serpin peptidase inhibitor, clade B, member 5	

*MCM7*	NM_005916	99	97	minichromosome maintenance deficient 7	

*SLC1A2*	NM_004171	99	96	Solute carrier family 1 member 2	glutamate transporter[1]

*HBB*	NM_000518	99	99	Hemoglobin, beta	

*PTGDS*	NM_000954	99	94	Prostaglandin D2 synthase 21 kDa	released from RPE during rod phagocytosis [17,19]

*T26536*	T26536	99	75	cDNA clone	

*EEF1A1*	NM_001402	99	80	Eukaryotic translation elongation factor 1 alpha 1	

*TTR*	NM_000371	99	80	Transthyretin (prealbumin, amyloidosis type I)	maintains normal levels of retinol and retinol binding proteins in plasma [14,20]

*BE260168*	BE260168	99	90	cDNA clone	

*CST3*	NM_000099	99	92	Cystatin C	associated with AMD[16,18]

*ZNF503*	NM_032772	99	95	Zinc finger protein 503	

*BG190000*	BG190000	99	94	cDNA clone	

*BE262306*	BE262306	99	80	cDNA clone	

*HSP90B1*	NM_003299	99	94	Heat shock protein 90 kDa beta (Grp94), member 1	[21]

*GNGT1*	NM_021955	99	90	Guanine nucleotide binding protein (G protein), gamma transducing activity polypeptide 1	

*RPL3*	NM_000967	99	20	Ribosomal protein L3	

*RPL41*	NM_021104	99	23	Ribosomal protein L41	

*AI857840*	AI857840	99	77	cDNA clone	

*PCSK7*	NM_004716	99	90	Proprotein convertase subtilisin/kexin type 7	

*AL521537*	AL521537	99	75	cDNA clone	

*CLU*	NM_001831	99	42	Clusterin	member of complement system [24]

Among these genes we identified five genes with known expression in the RPE (*SLC17A7*[14], *CST3*[18], *TTR*[14], *HSP90B1*[21] and *PTGDS*[17]). The *SLC17A7 *gene is a glutamate transporter, like the *SLC1A2 *gene which is also in the top 30 highly expressed genes. The CST3 gene was previously suggested to have an association with AMD[16,17]. The list also contains two genes with a role in the protection against oxidative stress (*MT1A*[22], *TP53*[23]). The *GNGT1 *gene, expressed in photoreceptors[30], suggests the inevitable presence of photoreceptor contamination. Perc: percentile.

#### Genes with moderate expression levels (μ_int _50^th^–90^th ^percentile)

Upon analyzing this group of 8,776 genes, we found a statistically significant overrepresentation of the Kegg pathways phosphatidylinositol signaling and aminosugars metabolism (Benjamini-Hochberg p value < 0.001) (Table 1).

#### Genes with low expression levels (μ_int _10^th^–50^th ^percentile)

Among the 8,776 genes with low expression levels there was a statistically significant overrepresentation of the neuroactive ligand-receptor interaction (Benjamini-Hochberg p value 0.001), long-term depression, O-glycan biosynthesis and calcium signaling pathways (Ease score p value < 0.001) (Table 1).

### Analysis of gene expression variability (CV)

We analyzed the interindividual variability in gene expression (CV) among the 19,746 genes with expression levels in the RPE higher than the 10^th ^percentile, (see Additional file 1: Expression level and interindividual variation in all genes on the custom microarray). Aside from the overrepresented cluster ECM-receptor interaction (Ease score p value < 0.001)(Table 1), this yielded little extra information compared to the CV assignment in subcategories of high, moderate and low expression levels (Table 1 and below), and is not presented in detail here. The thirty genes with the highest interindividual variation in expression levels in our dataset are presented in Table 3.

**Table 3:** The top thirty genes with the highest interindividual variation in expression levels (CV) between six healthy human donors, sorted descending by coefficient of variation (CV).

gene symbol	Genbank ID	mean intensity μ_int _perc	CV perc	gene name
*HSD17B2*	NM_002153	95	99	Hydroxysteroid (17-beta) dehydrogenase 2

*MYOC*	NM_000261	99	99	Myocilin, trabecular meshwork inducible glucocorticoid response

*OGN*	NM_014057	92	99	Osteoglycin (osteoinductive factor, mimecan)

*SFRP4*	NM_003014	93	99	Secreted frizzled-related protein 4

*AOC2*	NM_009590	91	99	Amine oxidase, copper containing 2 (retina-specific)

*DIO3*	NM_001362	85	99	Deiodinase, iodothyronine, type III

*SLC2A5*	NM_003039	62	99	Solute carrier family 2 (facilitated glucose/fructose transporter), member 5

*XIST*	AK025198	99	99	X (inactive)-specific transcript

*TFPI2*	NM_006528	99	99	Tissue factor pathway inhibitor 2

*CYR61*	NM_001554	98	99	Cysteine-rich, angiogenic inducer, 61

*FGFBP2*	NM_031950	84	99	Ksp37 protein

*FBP2*	NM_003837	64	99	Fructose-1,6-bisphosphatase 2

*EGFL6*	NM_015507	74	99	EGF-like-domain, multiple 6

*IL8*	NM_000584	66	99	Interleukin 8

*MFAP4*	L38486	99	99	Microfibrillar-associated protein 4

*CCL2*	NM_002982	90	99	Chemokine (C-C motif) ligand 2

*ZIC1*	NM_003412	67	99	Zinc family member 1 (odd-paired homolog, Drosophila)

*COL9A1*	NM_001851	88	99	Collagen, type IX, alpha 1

*CCL26*	NM_006072	79	99	Chemokine (C-C motif) ligand 26

*PITX2*	NM_000325	89	99	Paired-like homeodomain transcription factor 2

*ALDH1A1*	NM_000689	78	99	Aldehyde dehydrogenase 1 family, member A1

*HBG1*	NM_000559	75	99	Hemoglobin, gamma A

*S100A6*	NM_014624	99	99	S100 calcium binding protein A6

*IL6*	NM_000600	77	99	Interleukin 6 (interferon, beta 2)

*HBG2*	NM_000184	71	99	Hemoglobin, gamma G

*C13orf33*	NM_032849	93	99	Chromosome 13 open reading frame 33

*RBM3*	NM_006743	96	99	RNA binding motif (RNP1, RRM) protein 3

*CFB*	NM_001710	94	99	Complement factor B

*EGR1*	NM_001964	99	99	Early growth response 1

*PTX3*	NM_002852	97	99	Pentraxin-related gene, rapidly induced by IL-1 beta

Note that although *XIST, EGFL6 *and *RBM3 *are x-chromosomal transcripts, their high interindividual variation could not be explained by the gender of the donors (data not shown). Perc: percentile.

#### Genes with high interindividual variability (CV > 90^th ^percentile)

Among the 390 genes with both a high CV and high μ_int _there was an overrepresentation of genes involved in antigen processing as well as the complement and coagulation cascades. The 824 genes with a high CV and moderate μ_int _showed an overrepresentation of genes involved in focal adhesion and cytokine-cytokine receptor interaction, and the 762 genes with high CV and low μ_int _showed an overrepresentation of genes involved in type I diabetes mellitus. The latter group contains mainly major histocompatibility complex genes and *interleukin 1α *[genbank: NM_000575].

#### Genes with low interindividual variability (CV < 10^th ^percentile)

Table 4 shows the thirty genes with the most stable expression in macular RPE. Among the expressed genes (μ_int _> 10^th ^percentile) with stable RPE gene expression (CV < 10^th ^percentile, n = 1,972) there were no genes overrepresented in Kegg pathways. One hundred and ninety four of these 1,972 genes had high expression levels, 1,064 had moderate expression levels and 714 had low expression levels. Using the DAVID software, a significant overrepresentation of genes in the glycosaminoglycan degradation pathway was found in the group of 194 genes with stable expression and high expression levels (Ease score p value < 0.001) (Table 1).

**Table 4:** The thirty genes with the least interindividual variation in macular RPE gene expression levels among six healthy human donors, sorted ascending by coefficient of variation (CV).

gene symbol	Genbank ID	mean intensity μ_int _perc	CV perc	gene name
*EXOC3*	BC001511	85	< 1	Exocyst complex component 3

*PDXK*	AI571369	74	< 1	Pyridoxal (pyridoxine, vitamin B6) kinase

*ACY1*	NM_000666	63	< 1	Aminoacylase 1

*SS18L1*	AB014593	70	< 1	Synovial sarcoma translocation gene on chromosome 18-like 1

*FOSL1*	NM_005438	86	< 1	FOS-like antigen 1

*FPRL2*	NM_002030	56	< 1	Formyl peptide receptor-like 2

*CPSF4*	NM_006693	62	< 1	Cleavage and polyadenylation specific factor 4, 30 kDa

*FAM110B*	AK023658	40	< 1	Chromosome 8 open reading frame 72

*RAB20*	AW861333	20	< 1	Transcribed locus

*CHD2*	AW896069	15	< 1	Chromodomain helicase DNA binding protein 2

*BI001591*	BI001591	20	< 1	Transcribed locus

*FATE1*	NM_033085	37	< 1	Fetal and adult testis expressed 1

*CLEC4E*	NM_014358	44	< 1	C-type lectin domain family 4, member E

*PARN*	NM_002582	75	< 1	Poly(A)-specific ribonuclease (deadenylation nuclease)

*KIAA0586*	NM_014749	59	< 1	KIAA0586

*TMEM156*	NM_024943	41	< 1	Transmembrane protein 156

*CSNK2A1*	NM_001895	59	< 1	Casein kinase 2, alpha 1 polypeptide

*CGI-96*	NM_015703	35	< 1	CGI-96 protein

*LOC442100*	BM127012	26	< 1	Transcribed locus

*MYST3*	NM_006766	93	< 1	MYST histone acetyltransferase (monocytic leukemia) 3

*ZMYND8*	AF144233	33	< 1	Protein kinase C binding protein 1

*C20orf11*	AK025775	88	< 1	Chromosome 20 open reading frame 11

*GAK*	NM_005255	89	< 1	Cyclin G associated kinase

*SOBP*	NM_018013	67	< 1	hypothetical protein FLJ10159

*ZNF665*	NM_024733	16	< 1	Zinc finger protein 665

*BG742052*	BG742052	57	< 1	cDNA clone

*OR2A7*	AF327904	57	< 1	Olfactory receptor, family 2, subfamily A, member 7

*PRKCE*	NM_005400	18	<1	Protein kinase C, epsilon

*RNF14*	AB022663	88	< 1	Ring finger protein 14

*SELENBP1*	NM_003944	93	< 1	Selenium binding protein 1

Perc: percentile.

### Gene expression analysis of known retinal disease genes

#### Known macular disease genes

We then investigated both the expression levels and interindividual expression differences of 14 macular disease genes in our RPE gene expression dataset (Table 5).[26] In terms of expression levels, 63 percent of the macular disease genes were found in the top 10 percent of genes with high macular RPE expression levels. In terms of variability, 50 percent of the macular disease genes were found in the top 10 percent of genes with highly variable macular RPE expression levels. In addition, none of the macular degeneration genes were found in the 10 percent of genes with stable macular RPE expression.

**Table 5:** Expression levels and interindividual differences of currently known macular disease genes with RPE expression[26].

**gene symbol**	**Genbank accession**	**mean intensity μ_int _(perc)**		**CV (perc)**	
**high interindividual variation (CV > 10^th ^perc)**					

*CFB*	NM_001710	10,382	(94)	215	(99)

*C3*	NM_000064	9,910	(94)	145	(98)

*FBLN5*	NM_006329	2,823	(76)	136	(98)

*PRPH2*	NM_000322	32,204	(98)	86	(93)

*GUCA1B*	NM_002098	2,329	(72)	83	(93)

*CST3*	NM_000099	338	(26)	79	(92)

*CFH*	NM_000186	8,508	(92)	77	(91)

*TIMP3*	NM_000362	7,209	(91)	73	(90)

**intermediate interindividual variation (CV 10^th ^– 90^th ^perc)**					

*C2*	NM_000063	1,689	(65)	71	(90)

*BEST1*	NM_004183	132,611	(99)	58	(84)

*HTRA1*	NM_002775	45,420	(99)	47	(74)

*EFEMP1*	NM_004105	20,292	(97)	44	(70)

*C1QTNF5*	NM_015645	23,226	(98)	40	(64)

*TLR4*	NM_003266	225	(45)	38	(60)

**low interindividual variation (CV < 90^th ^perc)**					

none	-	-		-	

Data are grouped by coefficient of variation (CV) in descending order into three groups, high CV (> 90^th ^percentile, CV > 72), intermediate (CV 10^th ^– 90^th ^percentile) and low CV (< 10^th ^percentile, CV < 19). Perc: percentile.

A number of genes currently known or suggested to be associated with AMD, showed high (*C3 *[genbank: NM_000064], *CFB *[genbank: NM_001710], *CFH *[genbank: NM_000186], *HTRA1 *[genbank: NM_002775], and *CST3 *[genbank: NM_000099]) or moderate (*FBLN5 *[genbank: NM_006329]) expression levels in the RPE. With the exception of *HTRA1 *[genbank: NM_002775], all these genes also showed high interindividual variation.

#### Known peripheral retinal disease genes

Finally, we analyzed the gene expression levels and interindividual differences in expression of 93 genes known to be involved in diseases of the peripheral retina[26] in our macular RPE expression dataset (Table 6).

**Table 6:** Expression levels and interindividual differences of currently known peripheral disease genes with RPE expression[26].

**gene symbol**	**Genbank accession**	**mean intensity (μ_int_) (percentile)**		**CV (percentile)**	
**high interindividual variation (CV > 90^th ^percentile)**					

*COL9A1*	NM_001851	5,628	(88)	225	(100)

*RBP4*	NM_006744	20,291	(97)	196	(100)

*COL2A1*	NM_001844	527	(37)	122	(97)

*GNAT1*	NM_000172	100,271	(100)	80	(92)

*RDH5*	NM_002905	6,716	(90)	78	(92)

*RLBP1*	NM_000326	43,011	(99)	74	(91)

**intermediate interindividual variation (CV 10^th ^– 90^th ^percentile)**					

*PRCD*	AK054729	46,964	(99)	66	(88)

*GUCY2D*	NM_000180	2,672	(75)	64	(87)

*NPHP3*	AI200954	3,266	(79)	63	(86)

*IMPDH1*	NM_000883	12,177	(95)	63	(86)

*LRAT*	NM_004744	15,935	(96)	62	(86)

*LRP5*	NM_002335	519	(37)	62	(86)

*RD3*	AV721413	11,010	(95)	61	(86)

*RGR*	NM_002921	39,454	(99)	57	(83)

*TULP1*	NM_003322	6,284	(89)	57	(83)

*AHI1*	AL136797	7,168	(91)	56	(82)

*SEMA4A*	NM_022367	2,824	(76)	54	(80)

*TEAD1*	AL133574	4,473	(84)	51	(78)

*PANK2*	NM_024960	273	(20)	48	(75)

*PEX7*	NM_000288	1,734	(65)	48	(75)

*OAT*	NM_000274	3,762	(81)	45	(71)

*CDH3*	NM_001793	9,589	(93)	45	(71)

*BBS10*	NM_024685	1,556	(63)	45	(71)

*PRPF8*	NM_006445	6,510	(90)	40	(64)

*TIMM8A*	NM_004085	913	(50)	39	(63)

*FZD4*	NM_012193	7,964	(92)	39	(62)

*OPA3*	NM_025136	20,540	(97)	39	(62)

*COL11A1*	NM_001854	911	(50)	38	(61)

*PGK1*	NM_000291	12,114	(95)	37	(59)

*CYP4V2*	AK022114	5,411	(87)	37	(58)

*JAG1*	NM_000214	1,394	(60)	35	(53)

*MYO7A*	NM_000260	6,691	(90)	34	(53)

*BBS2*	NM_031885	4,446	(84)	34	(51)

*PAX2*	NM_003990	2,946	(77)	33	(50)

*BBS1*	NM_024649	3,750	(81)	32	(46)

*NYX*	NM_022567	372	(28)	32	(45)

*ABCC6*	NM_001171	1,555	(63)	28	(35)

*MERTK*	NM_006343	4,763	(85)	28	(35)

*ARL6*	BI914103	1,023	(53)	28	(34)

*MFRP*	NM_031433	13,200	(96)	27	(33)

*PXMP3*	NM_000318	5,341	(87)	25	(25)

*ALMS1*	AB002326	962	(51)	23	(20)

*MKKS*	NM_018848	2,056	(69)	22	(16)

*NDP*	NM_000266	1,335	(59)	20	(14)

*PHYH*	NM_006214	3,293	(79)	20	(12)

*WFS1*	NM_006005	4,935	(86)	19	(10)

**low interindividual variation (CV < 10^th ^percentile)**					

*PRPF31*	NM_015629	3,187	(78)	17	(06)

*PEX1*	NM_000466	3,008	(77)	15	(04)

*PRPF3*	NM_004698	7,057	(91)	14	(03)

*TRIM32*	NM_012210	2,277	(71)	13	(02)

*CLN3*	NM_000086	2,330	(72)	8	(0)

Data are grouped by coefficient of variation (CV) in descending order into three groups, high CV (> 90^th ^percentile, CV > 72), an intermediate group (CV 10^th ^– 90^th ^percentile) and CV low (< 10^th ^percentile, CV < 19).

Of this group, 32 percent were found in the 10 percent of genes with high expression levels in the macular RPE. Eleven percent of the known peripheral disease genes were found in the 10 percent of genes with high interindividual variation in expression in the macular RPE.

## Discussion

This study presents the first comprehensive analysis of the macular RPE transcriptome, with a focus on interindividual differences in RPE gene expression levels. We based our analyses on microarray data from six healthy human donor eyes. In addition, we performed a Kegg pathway analysis on genes with high, moderate and low expression levels and on genes with high and low interindividual variation in expression.

Only five genes from our top 30 most highly expressed RPE genes were previously known to be expressed in the human RPE *in vivo*: *SLC17A7 *[genbank: NM_020309][14], *CST3 *[genbank: NM_000099])[18], *PTGDS *[genbank: NM_000954][17], *TTR *[genbank: NM_000371][14]and *HSP90B1 *[genbank: NM_003299])[21] illustrating the lack of knowledge on the RPE transcriptome.

### Strengths and limitations of the study design

A recent statistical review suggested that a microarray study investigating a single tissue type, requires 6 biological replicate samples to draw statistically significant conclusions.[27] Consequently, we used the RPE gene expression from 6 different individuals. Previous RPE gene expression studies were based on less than six eyes, with the exception of a single cDNA microarray study limited to 4,325 genes that was based on 15 individuals. [8-12]

**Figure 3:**
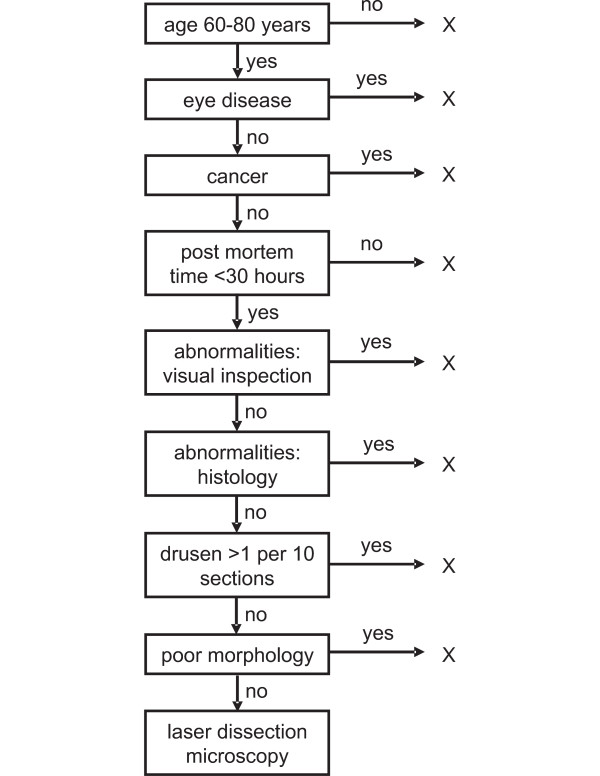
**Flow diagram of criteria used for the selection of donor eyes**. Donor eyes were required to meet all selection criteria before inclusion in the study. X indicates exclusion from the study. Donors were all between 60 and 80 years old in order to exclude the presence of undetected monogenic disorders. The presence of any known eye disease or malignancy was used as an exclusion criterion since both can alter (RPE) gene expression levels. Post mortem times were required to be less than 30 hours to reduce the effects of RNA degradation. Ocular abnormalities on visual or histological inspection served as exclusion criteria, specifically any signs of early AMD, defined by us as the presence of more than 1 druse per 10 histological sections. Poor morphology of the retina was also an exclusion criterion.

Our study design has a number of strong points and limitations, previously described in detail.[13] In summary, the strength of our study design comes from our strict selection criteria for the donor eyes (see Figure 3), the use of a laser dissection microscope for high cellular specificity and minimal tissue manipulation, large scale analysis using a 22 k microarray and a common reference design for comparison of all samples. Overall, our study was designed to minimize gene expression differences due to sampling methodology (see Figure 3) and technical causes, avoiding unnecessary mechanical handling of the freshly frozen tissue, the use of laser dissection microscopy to isolate homogeneous cell samples, stringent control of RNA quality and amplification procedures.[9,11-13] Initially, we performed dye swap experiments as technical replicates for three of our samples in order to ascertain the potential variability induced by dye bias. We observed a high correlation between the data from our analysis including and excluding the dye swap experiment (data not shown). At the same time, Dobbin (2003), Simon (2003) and others, used a similar study design as we did, and concluded that in a common reference design it is not necessary to perform dye swaps if the common reference is consistently labeled with the same dye. [28,29] Potential gene-specific dye bias will affect all experimental samples equally, and therefore does not confound the comparisons.[27] Consequently, we decided to perform the remaining three experiments without a dye swap.

One of the methodological limitations of our study was the limited number of eyes that met our selection criteria. The availability of a larger number of eyes would render more robust results with regard to interindividual variation. Nonetheless, our data give a good first impression of variability in gene expression levels in the RPE. An additional limitation is that a small amount of photoreceptor contamination was inevitably present in our RPE sample, see also table 2.[8,13,30] Furthermore, we cannot distinguish possible transient from permanent gene expression level differences. Our study is also limited by the fact that the measurement of gene expression of individual genes by microarray is inevitably influenced by a number of factors, like oligo design and the continuous updates of the human genome sequence. To correct for this last limitation, we focused our analysis on groups of genes with a wide range of expression levels, rather than on individual gene expression levels. Finally, our cut off criteria for high and low expression levels and interindividual differences are arbitrary. While this may indeed have consequences for individual genes, the impact on our functional analysis, which is based on large numbers of genes, will be minimal.

Despite these limitations, our data, combined with data from other retinal gene expression studies (which use a range of techniques, like SAGE and RT-PCR, that bear their own limitations),[13,31] contributes significantly to the currently expanding knowledge of the RPE transcriptome.

### Functional assessment of native gene expression in the macular RPE

The notion that the identity of a cell type is determined by the genes it expresses, prompted us to analyze the native macular RPE transcriptome. In the following section we describe the overrepresented functional groups that we identified in the RPE.

#### Highly expressed RPE genes and oxidative stress

Both functional annotation with DAVID and Ingenuity analysis independently indicate a statistically significant overrepresentation of genes associated with oxidative phosphorylation and ATP synthesis in our dataset. This is in line with the fact that the RPE has a high metabolic activity and energy demand. The down side of this high activity is that the RPE has to deal with large amounts of oxidative stress. The oxidative stress in the RPE is further augmented by the light projected onto the retina combined with the rich oxygen supply and lipid peroxidation in phagocytosed rod outer segments.[1,32] Given the high level of oxidative stress, the expression of genes contributing to the defense of the RPE cell against oxidative stress is essential for cell survival. Our data confirm this notion, which is highlighted by the expression of the *MT1A *[genbank: K01383] gene, a metallothionein, and the *TP53 *[genbank: NM_000546] gene in the top 30 most highly expressed RPE genes. Metallothioneins are thought to play a role in protection against oxidative stress; addition of *TP53 *[genbank: NM_000546] to human cell lines leads to a 50 percent decrease in reactive oxygen species.[22,23]

#### RPE and the immune system

Our data show an overrepresentation of genes with highly variable expression in a number of pathways related to the immune system. We identified the following four pathways, the complement and coagulation cascades (high expression levels), the antigen processing and presentation pathway (high expression levels) and the cytokine-cytokine receptor interaction pathway (moderate expression levels). Both the antigen processing and presentation pathway and the type 1 diabetes mellitus pathway contain MHC genes responsible for antigen presentation. Cytokine production is highly sensitive to inflammation in the RPE.[33] The cytokine-cytokine receptor pathway contains a number of chemokines, small secreted proteins involved in the chemotaxic attraction of monocytes and neutrophils. The highly variable expression of genes involved in the immune system is most likely explained by both genetic differences and a variable degree of subclinical inflammation (local or systemic) among our donors.

#### RPE genes and the extracellular matrix (Bruch's membrane)

The close interaction of the RPE with Bruch's membrane (BM) is exemplified by the overrepresentation of genes in two pathways. The first pathway contains genes involved in extracellular matrix (ECM) receptor interaction.[13] The ECM receptor interaction pathway, part of the focal adhesion pathway, contains *collagens type I, III and IV, thrombospondin, laminin beta 1 *[genbank: NM_002291], *fibronectin 1 *[genbank: NM_002291], *reelin *[genbank: NM_005045], and *cd44 antigen *[genbank: NM_000610]. *Collagen type IV, laminin *and *fibronectin *are all main components of basement membranes, such as BM. Surprisingly, the genes in this group showed highly variable expression, which may indicate that the molecular composition of BM is different among individuals. Alternatively, it has been described that with age, the solubility of collagens in BM decreases significantly.[34] Thus, the high variability in expression levels of collagen genes between our samples can perhaps be explained by differences in the physiological donor age.

A second pathway that connects RPE expressed genes to BM is the glycosaminoglycan (GAG) degradation pathway. There was an overrepresentation of genes with stable and high expression in this pathway. GAG synthesis has been shown in cultured RPE and GAG's are secreted into the extracellular matrix and BM.[35] Interestingly, GAG's are rapidly turned over in the RPE, and the composition of GAG in BM changes with age. [35-37] Our data suggest there is a strict regulation of GAG turnover in the RPE, even in donors of different ages.

#### Additional RPE gene functions

In addition to the involvement of the RPE genes in oxidative stress, BM and the immune system, analysis of our data revealed the following two functional categories: protein synthesis and glutamate transport.

A high level of protein synthesis is essential for the RPE to maintain its multiple functions.[1] This is exemplified by the overrepresentation of genes with high expression in the ribosomal protein activity pathway.

Glutamate transport is an important process in the RPE. The top 30 most highly expressed RPE genes contained two glutamate transporters *SLC1A2 *[genbank: AF131756] and *SLC17A*7 [genbank: AF131756]. The latter transporter was already known to be expressed in the human RPE *in vivo*.[14,38] Glutamate is an important neurotransmitter that is released from the photoreceptors both in a light influenced fashion, and upon apoptosis. Since high concentrations of glutamate are neurotoxic, re-uptake and transport of glutamate are essential for the normal retinal homeostasis.[38]

Finally, in the overlap between previous RPE studies[14] and genes with high expression in our RPE transcriptome, we identified the cell-cell signaling and interaction network. This network contains several genes involved in signal transduction, like *SLC7A2 *[genbank: AL512749] and *NCK2 *[genbank: BC007195] further emphasizing the important role of the RPE in interaction with other cell types.[39,40]

### Comparison with literature

Comparison of our most highly expressed RPE genes to the literature revealed a distinct overlap. Schulz and coworkers recently combined different analyses of the retina/RPE/choroid transcriptome, and described 13,000 retina/RPE genes found in at least two studies.[14] Out of these 13,000, we currently assign 7,231 genes to be expressed by the RPE, 1,407 of which are highly expressed. (see Additional file 2: overlap between highly expressed RPE genes and retina/RPE genes in at least two studies) In addition, the same review[14] suggested that 246 genes were expressed only in RPE studies. We assign 137 of these 246 genes to the RPE as well; 17 out of these 137 genes have high expression levels in our RPE transcriptome analysis. (see Additional file 3: overlap between highly expressed RPE genes and genes found only in RPE studies)

Finally, of the genes previously described to be specifically expressed either in the retina or the RPE in individual studies,[14] 39 genes are also present in our RPE transcriptome analysis. Twenty two of these 39 genes had high expression levels. (see Additional file 4: overlap between highly expressed RPE genes and retina/RPE genes in single studies)

While data on interindividual variation in RPE gene expression are lacking, functional properties of RPE genes have been investigated previously.

The combined functional annotation from three studies resemble our functional annotation in the following areas: gene regulation, transcription, protein metabolism, cell proliferation, survival and signaling, energy metabolism, cytoskeleton and inflammation.[8,10,11] The current study adds the following more specific functional categories, oxidative phosphorylation, ATP synthesis, ribosome, phosphatidylinositol signaling and aminosugars metabolism. Among the highly expressed RPE genes we identified an overrepresentation of the complement cascade and genes involved in the composition of BM.

### Gene expression analysis of known retinal disease genes

In our macular RPE sample we observed that 63 percent of genes involved in macular disorders according to the literature,[26] had high expression levels. In contrast, only 32 percent of the peripheral retinal disease genes[26] were highly expressed in our sample. These figures may be biased, since the search for candidate genes has been focused on cell-specific highly expressed genes in the first place. The figures probably reflect the fact that RPE gene expression differences exist between the retinal macula and the periphery.[13] However, our data probably also imply that the mean expression level of a gene in the RPE is informative in the search for new candidate disease genes.

With respect to the variability in gene expression, we found that the interindividual differences of currently known macular retinal disease genes were somewhat higher than the overall pattern of variation seen in the entire array. Whether or not this finding is coincidental remains to be elucidated.

## Conclusion

In conclusion, we present comprehensive data on (interindividual differences of the) gene expression profile of the RPE based on 22,000 genes from six different healthy human donors. This is the first study to describe the interindividual variability in gene expression levels from a microarray analysis of the RPE transcriptome.

There was no correlation between the height of gene expression (μ_int_) and the interindividual variability (CV) (data not shown). We noted a more than hundred fold difference in CV between genes with stable expression and genes with variable expression levels.

Our data show that the RPE most likely has high levels of protein synthesis, a high energy demand and is subject to high levels of oxidative stress as well as a variable degree of inflammation. Finally, our data show high interindividual variability in expression of ECM genes and indicate a high and constant level of glycosaminoglycan (GAG) turnover, two functions related to BM.

The fact that large interindividual differences exist in the expression of a number of known retinal disease genes has not only functional implications, but is also relevant for new candidate disease gene identification and the development of dose-dependent (gene) therapeutic strategies.

## Methods

### Human donor eyes

This study was performed in agreement with the declaration of Helsinki on the use of human material for research. Material used in this study was provided to us by the Corneabank Amsterdam. In order to minimize genetic heterogeneity, we selected six eyes from a total of 200 human donor eyes using strict selection criteria, (see Figure 3). In summary, donors were excluded when their age was not between 60 and 80 years, when they had an eye disease or any form of malignancy and when the time between death and enucleation of the eye was more than 30 hours. Furthermore, eyes were excluded when they showed any abnormalities upon visual or histological examination: more specifically, when more than one druse was seen in 10 histological sections, or when retinal morphology was poor. All donors were Caucasian, five were male, one was female. The donors died of cardiovascular or cerebrovascular causes or of chronic obstructive pulmonary disease. Donors did not have a known ophthalmic disorder or malignancy. Globes were enucleated between 14 and 27 hours post mortem and frozen several hours later according to a standard protocol. Donors were aged 63 to 78 years at the time of death. We chose old donors in order to minimize the likelihood of the presence of yet undiagnosed monogenic eye diseases. This does not rule out the presence of the most common retinal disease in the old eye, age related macular degeneration (AMD). Therefore the donor retinas were thoroughly screened for early signs of AMD by histological examination (the presence of more than 1 druse in 10 sections). Visual examination and histological examination, including periodic acid Schiff (PAS) staining, indicated no retinal pathology in any of the donor eyes.

### RPE cell sampling

Globes were snap-frozen and stored at -80°C until use. A macular fragment of 16 mm^2 ^with the fovea in its center was cut from each of the retinas, as described previously.[13] In summary, for each eye, 10 cryosections, 8 μm thick, spaced no more than 220 μm apart were stained with periodic-acid Schiff and microscopically examined for abnormalities, such as drusen indicative of early-AMD.

Twenty μm sections from the macular areas were used for the isolation of RPE cells. These sections were dehydrated with ethanol and air-dried before microdissection with a Laser Microdissection System (PALM, Bernried, Germany) using a pulsed laser. A total of up to 10,000 RPE cells per eye were microdissected and stored at -80° Celsius.

### RNA isolation and (single) amplification

Total RNA was isolated and the mRNA component was amplified essentially as described previously.[13] Next, the amplified RNA (aRNA) samples were quantified with a nanodrop (Isogen Life Science B.V., The Netherlands) and the quality was checked on a BioAnalyzer (Agilent Technologies, Amstelveen, The Netherlands). Subsequently, aRNA samples were labeled with either a Cy3 or a Cy5 fluorescent probe.

### Microarray handling

A common reference design was applied in our microarray hybridizations using the common reference sample described in the study of van Soest *et al *(2007).[13] In summary, the common reference sample consists of aRNA from a pool of RPE/choroid isolated from 10 donor eyes (mean age 60 years). aRNA from all six donors and the common reference sample was labeled. Subsequently, labeled aRNA from the donors was hybridized against the common reference sample to six 22 k custom arrays. Initially, a dye swap experiment was performed for three of the six donor samples in order to assess potential variability introduced by dye-bias for methodological reasons (see discussion). Dye swaps were disregarded in the final analysis. Arrays were enriched for sequences expressed in RPE, neural retina and brain (Agilent Technologies, Amstelveen, The Netherlands), (see Additional file 1: Expression level and interindividual variation in all genes on the custom microarray). Hybridization, washing and scanning were performed as described previously.[13]

### Data analysis

**Figure 4:**
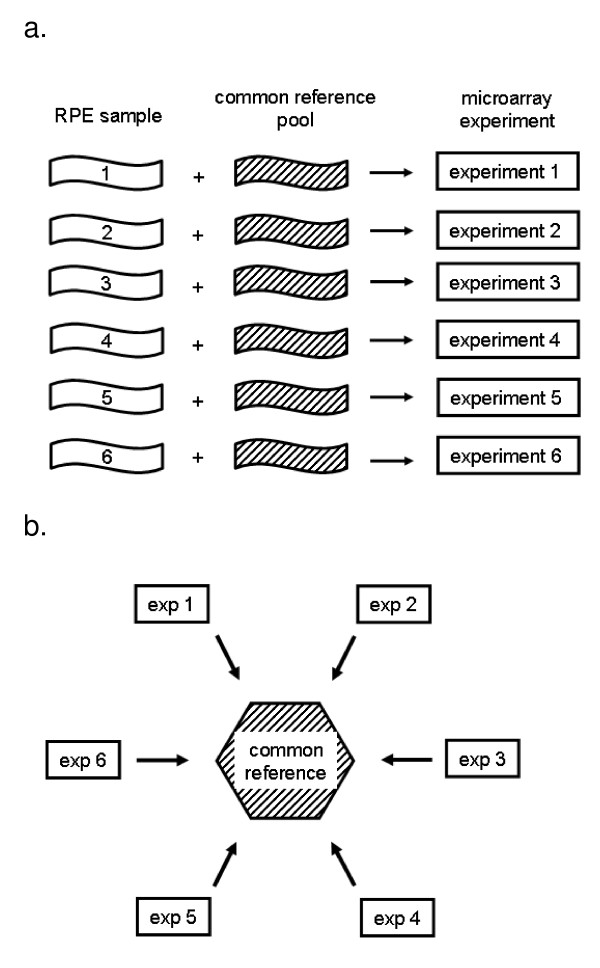
**Study design**. A. Experimental setup. Six RPE samples from 6 different donors were hybridized to six microarrays along with the common reference sample. B. Data analysis. The common reference was used to normalize the RPE expression data from the six arrays which enabled comparison of the six individuals.

Scanned images were processed with Feature Extraction software (v 8.5 Agilent). Data from all six hybridizations was analyzed with Rosetta Resolver software (Rosetta Inpharmatics). The signal of each of the six RPE samples was normalized using the common reference sample. This enabled a direct comparison of the six RPE samples (Figure 4). We used six biological replicates in order to draw significant conclusions.[27] For each gene we calculated the mean signal intensity (μ_int_) and standard deviation (σ) of the six biological replicates. While a limited number of genes is present on the array more than once, for the analyses of large groups of genes we regarded the number of entries on the array equal to the number of genes. Genes were grouped according to their mean intensity (μ_int_). We defined μ_int _above the 90^th ^percentile as high expression, μ_int _between the 90^th ^and 50^th ^percentile as moderate expression and μ_int _between the 50^th ^and the 10^th ^percentile as low expression. We considered the genes in these three groups to have potential biological significance. Genes with a μ_int _below the 10^th ^percentile were considered to have very low expression with a doubtful biological significance.

In order to describe the interindividual differences in gene expression levels between all six eyes systematically, we calculated the coefficient of variation (CV), defined as the standard deviation divided by the mean (σ/μ_int_), for each gene. We considered genes with a CV above the 90^th ^percentile to have "high" interindividual variation in expression and genes with a CV below the 10^th ^percentile to have "low" interindividual variation, or stable expression. Obviously, the categories for intensity and variability of expression were chosen somewhat arbitrarily, but they were essential to facilitate systematic analysis and to minimize the number of false positive results.

A functional analysis of Kegg pathways (Kyoto Encyclopedia of Genes and Genomes) was performed on genes with high, moderate and low expression levels and on genes with high and low interindividual variation using the DAVID online software.[41] Cut off criteria used were a p-value of less than 0.001 using either a Benjamini-Hochberg correction or an Ease score, which is a modified Fisher's exact test[41,42].

We compared our RPE transcriptome to a compilation of the mammalian retina/RPE transcriptome, which is based on multiple independent gene expression studies of combinations of the neural retina/RPE/choroid in the literature[14]. Overlap between the two datasets was analyzed using Ingenuity Pathways Analysis (Ingenuity^® ^Systems) resulting in a connectivity network describing the underlying biology of RPE cells at the genomic and proteomic level[43].

## Authors' contributions

JB performed microarray analysis, bioinformatic analyses, drafted and finalized the manuscript together with AB. SvS supervised sample selection and preparation, microarray analysis and critically read and commented on the manuscript. SS performed Ingenuity analyses and critically read and commented on the bioinformatics as well as the manuscript. AE carried out extensive sample preparation, optimized and assisted in technical procedures, read and commented on technical aspects of the manuscript. AV daily supervision bioinformatic Ingenuity analysis, critically read and commented on bioinformatics strategy as well as the manuscript. PS assisted with the design of the bioinformatics, provided funds and access to Ingenuity, and critically read and commented on the manuscript. TG assisted in the design of the study and experiments and critically read and commented on the manuscript. AB conceived the study, participated in its design and coordination, provided funds, drafted and finalized the manuscript together with JB.

## Supplementary Material

Additional file 1 **Expression level and interindividual variation in all genes on the custom microarray.** Click here for file

Additional file 2 **Overlap between highly expressed RPE genes and retina/RPE genes in at least two studies.** Click here for file

Additional file 3 **Overlap between highly expressed RPE genes and genes found only in RPE studies.** Click here for file

Additional file 4 **Overlap between highly expressed RPE genes and retinaRPE genes in single studies** Click here for file
